# Supplementary figures and images for: Modulation of Cisplatin Sensitivity through TRPML1-Mediated Lysosomal Exocytosis in Ovarian Cancer Cells: A Comprehensive Metabolomic Approach
Source: Cells. 2024 Jan 8;13(2):115. doi: 10.3390/cells13020115 (PMC10814698; doi:10.3390/cells13020115)

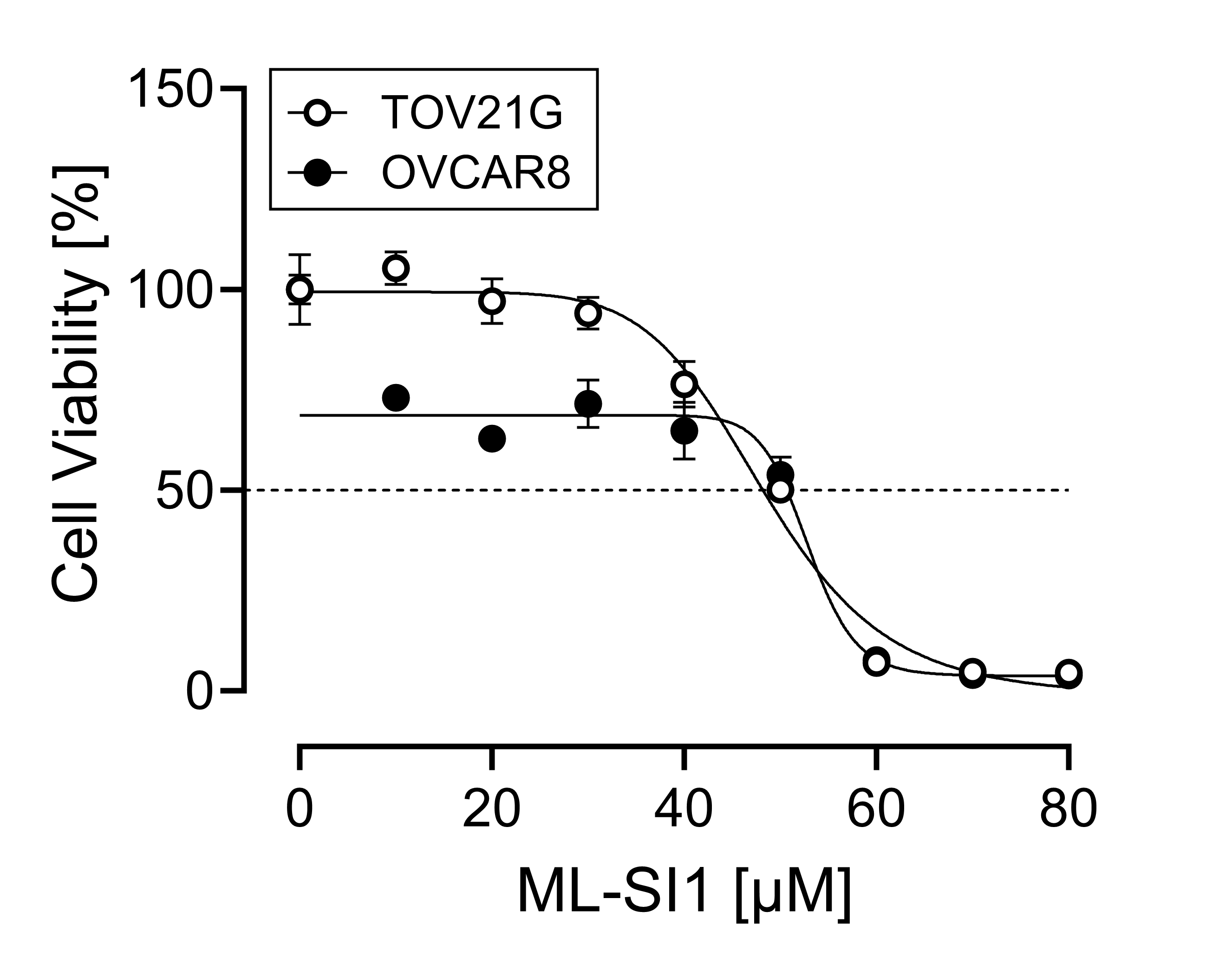

Supplement: Supplementary file 1 [file cells-13-00115-s001.zip › cells-2772757-supplementary.tif]
